# PIEZO1 Mediates Apoptosis of Endothelial Cells via Enhancing HMGA2 Expression Under Simulated Microgravity

**DOI:** 10.3390/ijms27031425

**Published:** 2026-01-30

**Authors:** Yuan Wang, Ruonan Wang, Xiaodong Qin, Yikai Pan, Chengfei Li, Xiqing Sun

**Affiliations:** Department of Aerospace Medical Training, School of Aerospace Medicine, Air Force Medical University, Xi’an 710032, China; wangcircle22@163.com (Y.W.); xiaopinannan@126.com (R.W.); xdqin1991@163.com (X.Q.); panyk0820@163.com (Y.P.)

**Keywords:** simulated microgravity, PIEZO1, HMGA2, Ca^2+^, apoptosis

## Abstract

Exposure to microgravity results in cardiovascular deconditioning, with endothelial cell apoptosis recognized as a pivotal initiating event. However, the mechanosensitive mechanisms underlying this process remain poorly understood. Here, we demonstrate that the expression of mechanosensitive ion channel protein PIEZO1 is upregulated in human umbilical vein endothelial cells (HUVECs) under simulated microgravity. Functional studies revealed that PIEZO1 activation promotes endothelial apoptosis under simulated microgravity conditions. Proteomic analysis following PIEZO1 knockdown revealed extensive alterations in biological processes associated with apoptosis. Furthermore, we found that PIEZO1 activation triggers calcium influx, leading to elevated expression of the HMGA2. Moreover, we identify that PIEZO1 activation induces calcium influx, which subsequently elevates the expression of HMGA2. The knockdown of HMGA2 significantly mitigated microgravity-induced endothelial apoptosis, indicating its role in PIEZO1-mediated apoptosis. These findings reveal a novel PIEZO1–Ca^2+^–HMGA2 axis critical for microgravity-induced endothelial apoptosis, providing mechanistic insight into cardiovascular adaptation to spaceflight and potential therapeutic targets for countermeasure development.

## 1. Introduction

Exposure to microgravity induces profound physiological adaptations across multiple organ systems, including muscle atrophy [1], bone demineralization [2], immune system dysregulation [3], and cardiovascular impairment [4]. Under microgravity conditions, the cardiovascular system experiences considerable unloading, characterized by reduced circulatory blood volume, decreased diastolic blood pressure, diminished left ventricular mass, and impaired cardiac contractility [5]. These hemodynamic alterations contribute to post-flight orthostatic intolerance, substantially compromising astronauts’ operational capacity upon re-entry into gravitational environments. Emerging evidence suggests that microgravity-induced endothelial cell dysfunction may play a pivotal role in cardiovascular deconditioning [6]. Our previous research has demonstrated that microgravity induces apoptosis in Endothelial cells (ECs), suggesting that apoptosis may play a pivotal role in the pathophysiological changes observed in the cardiovascular system [7]. At present, the precise mechanism underlying microgravity-mediated endothelial dysfunction remains to be elucidated.

ECs constitute a monolayer of epithelial cells that line the inner layer of the vascular lumen vessel wall, playing essential roles in maintaining vascular homeostasis. These roles include regulating vascular tension, thrombotic balance, smooth muscle cell proliferation, and inflammatory responses [8]. As key indicators of vascular health, endothelial cells are particularly susceptible to hemodynamic changes, with apoptosis serving as a fundamental mechanism of vascular remodeling. Apoptosis, a programmed form of cell death, facilitates tissue remodeling during development by eliminating superfluous cells [9]. While programmed cell death facilitates physiological tissue restructuring during development, including hyaloid vessel regression [10] and angiogenic pruning [11], dysregulated endothelial apoptosis under pathological conditions can initiate vascular dysfunction. Endothelial apoptosis has been implicated as an initiating factor in cardiovascular deterioration [12]. However, a comprehensive elucidation of the underlying molecular pathways under simulated microgravity is lacking.

ECs possess the ability to detect mechanical forces, particularly shear stress, and convert these forces into biological signals via various membrane proteins. The mechanosensitive cation channel PIEZO1 has emerged as a critical force sensor in endothelial cells [13,14]. It has been implicated in various endothelial functions, including eNOS phosphorylation and regulation of vascular endothelial protein tyrosine phosphatase (VE-PTP) [15]. Our recent work revealed that PIEZO1 mediates microgravity-induced endothelial migration through CXCR4 upregulation [16], suggesting its broader involvement in gravitational stress responses. Despite these advances, the specific contribution of PIEZO1 to microgravity-associated endothelial apoptosis remains unexplored.

This study aims to investigate the role of PIEZO1 in mediating endothelial cell apoptosis under simulated microgravity conditions and to elucidate the underlying molecular mechanisms using in vitro models. This work will provide novel insights into the mechanisms of vascular deconditioning induced by microgravity and may identify potential therapeutic targets for mitigating the adverse vascular effects associated with spaceflight.

## 2. Results

### 2.1. Simulated Microgravity Increases the Expression of PIEZO1 in HUVECs

**Figure 1:**
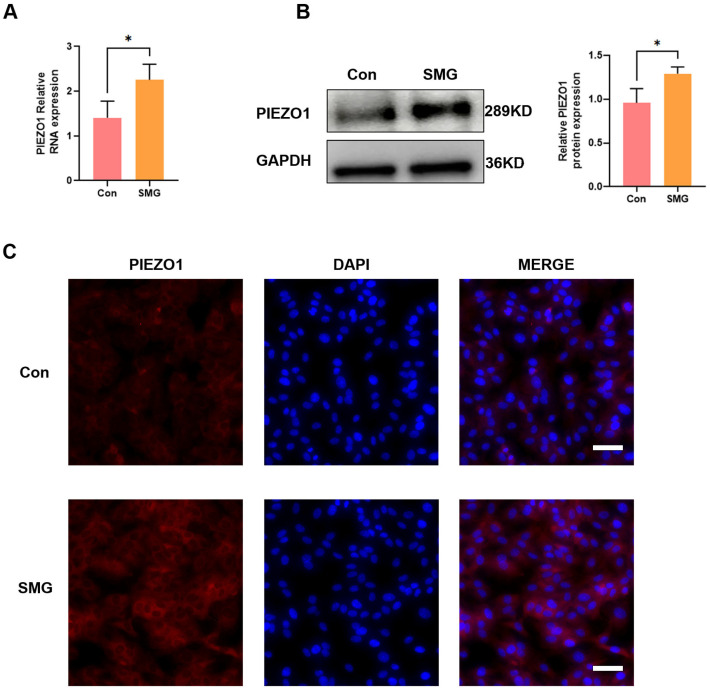
Simulated microgravity promotes PIEZO1 expression in HUVECs. (**A**) qRT-PCR analysis of PIEZO1 in HUVECs after simulated microgravity for 48 h. (**B**) Western blot analysis for the expression of PIEZO1 in HUVECs after simulated microgravity for 48 h. (**C**) The immunofluorescence staining of PIEZO1 (red immunofluorescence) and DAPI (blue immunofluorescence). (The data represent the mean ± *SEM*, *n* = 3; ns (no significance), *p* > 0.05; * *p* < 0.05, Scale Bar = 50 μm).

In this study, we conducted an in vitro experiment to investigate the effect of simulated microgravity on PIEZO1 expression in HUVECs. The cells were subjected to clinorotation for 48 hto mimic the effects of microgravity. We employed quantitative real-time PCR (qRT-PCR) and Western blot analysis to assess the expression levels of PIEZO1 at both the mRNA and protein levels. As depicted in Figure 1A, the mRNA expression of PIEZO1 was significantly upregulated after 48 h of clinorotation. Similarly, Figure 1B illustrates that the protein expression of PIEZO1 also increased under the same conditions. Furthermore, immunofluorescence assays confirmed the upregulation of PIEZO1 in HUVECs following 48 h of simulated microgravity, which corroborated the findings from the qRT-PCR and Western blot analyses (Figure 1C). Collectively, these data suggest that PIEZO1 expression is upregulated in HUVECs under conditions of simulated microgravity.

### 2.2. PIEZO1 Contributes to Simulated Microgravity-Induced Endothelial Cell Apoptosis

**Figure 2:**
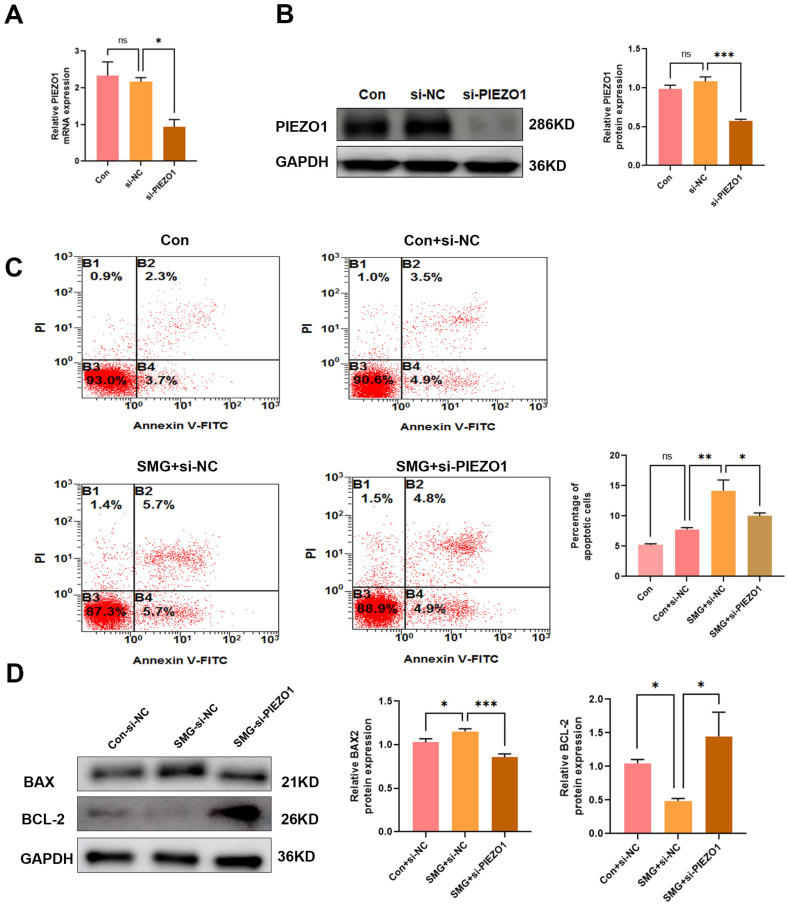
Simulated microgravity triggers endothelial cell apoptosis through PIEZO1 activation. (**A**) The knockdown efficiency of PIEZO1 in HUVECs was validated by qRT-PCR. (**B**) PIEZO1 protein knockdown in HUVECs was further confirmed by Western blot. (**C**) Analysis of cell apoptosis in HUVECs using Annexin V and PI staining through flow cytometry. Con+si-NC (control group transfected with negative control siRNA), SMG+si-NC (Simulated microgravity-treated group transfected with negative control siRNA), SMG+si-PIEZO1 (Simulated microgravity-treated group transfected with PIEZO1 siRNA). (**D**) Analysis of apoptosis-related proteins in HUVECs using Western blot. (The data represent the mean ± *SEM*, *n* = 3; ns (no significance), *p* > 0.05; * *p* < 0.05, ** *p* < 0.01, *** *p* < 0.001).

Endothelial cell apoptosis is crucial for vascular growth and remodeling [17]. Our previous study demonstrated that simulated microgravity induces apoptosis in endothelial cells [7]. To investigate the role of PIEZO1 in this process, we silenced PIEZO1 expression in HUVECs using a gene-specific siRNA. Knockdown efficiency was verified as previously described [16], with representative results shown in Figure 2A,B. We then analyzed apoptosis in HUVECs under simulated microgravity (48 h) and normal conditions using flow cytometry and Western blot analysis. Flow cytometry analysis demonstrated that simulated microgravity led to an increased apoptosis rate in HUVECs. Notably, apoptosis was reduced in PIEZO1-knockdown cells compared to SMG+siNC-transfected cells (Figure 2C). Consistent with these findings, Western blot analysis demonstrated upregulation of pro-apoptotic Bax and downregulation of anti-apoptotic Bcl-2 in simulated microgravity-exposed cells compared to Con+si-NC group (Figure 2D). Notably, PIEZO1 depletion attenuated these apoptotic alterations (Figure 2D). These findings suggest that microgravity induces the apoptosis of ECs through PIEZO1.

### 2.3. Silencing of PIEZO1 Alters Diverse Biological Processes in HUVECs Under Simulated Microgravity

**Figure 3:**
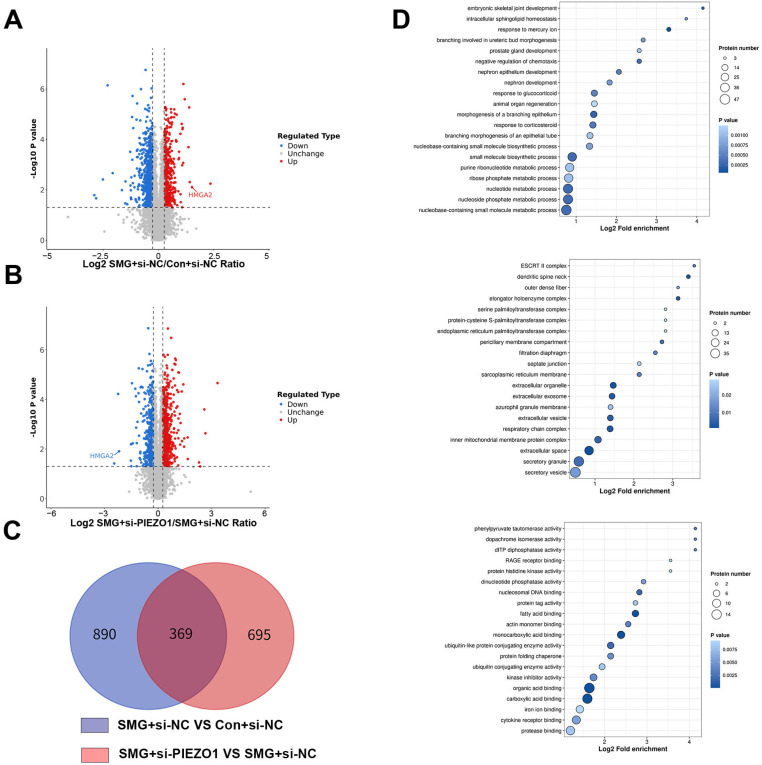
The proteomics of HUVECs after knockdown of PIEZO1 under simulated microgravity. (**A**,**B**) Volcanic maps of differential proteins, where red dots indicate significant upregulation, blue dots indicate significant downregulation, and redindicates no significant differences. (**C**) A Venn diagram compared the significant DEPs of SMG+si-NC vs.Con+si-NC and SMG+si-PIEZO1 vs. SMG+si-NC. (**D**) The GO analysis of DEPs. (*n* = 3).

To elucidate the molecular mechanisms underlying PIEZO1’s regulation of endothelial cell apoptosis under simulated microgravity, we conducted a proteomic analysis of HUVECs using liquid chromatography-tandem mass spectrometry (LC-MS/MS). We collected samples from three groups: Con+si-NC SMG+si-NC and SMG+si-PIEZO1, for total protein extraction and subsequent proteomics analysis. The high repeatability among samples within each group, as demonstrated by principal component analysis, has been confirmed in previous studies [16]. A stringent criterion was employed for quantitative analysis, with a fold-change threshold set at >1.2 for significant upregulation and <1/1.2 for downregulation (*p* < 0.05). Comparative LC-MS/MS analysis between the SMG+si-NC and Con+si-NC groups revealed 1259 differentially expressed proteins (DEPs), with 776 downregulated and 483 upregulated (Figure 3A). Similarly, comparison between the SMG+si-PIEZO1 and SMG+si-NC groups identified 1064 DEPs, including 490 downregulated and 574 upregulated proteins (Figure 3B).

A Venn diagram comparison of significant DEPs between SMG+si-NC vs. Con+si-NC and SMG+si-PIEZO1 vs. SMG+si-NC revealed 369 overlapping proteins (Figure 3C, Supplementary Table S1). To investigate the proteomic changes induced by PIEZO1 under simulated microgravity, we performed Gene Ontology (GO) enrichment analysis on the 369 overlapping proteins. Biological processes such as “nucleobase-containing small molecule metabolic process” “nucleoside metabolic process,” and “nucleoside phosphate metabolic process.” In molecular functions, it was enriched for “protease binding,” “cytokine receptor binding,” and “iron ion binding.” In cellular components, it included “secretory vesicle,” “secretory granule,” and “extracellular space” (Figure 3D).

Our analysis identified HMGA2, a key regulator of endothelial cell function, which was found to be upregulated in SMG+si-NC vs. Con+si-NC but downregulated in SMG+si-PIEZO1 vs. SMG+si-NC. HMGA2 was particularly enriched in “regulation of apoptotic process,” leading us to hypothesize that HMGA2 may play a predominant role in PIEZO1-mediated endothelial cell apoptosis under simulated microgravity conditions.

This refined analysis provides a clearer understanding of the proteomic landscape influenced by PIEZO1 in HUVECs under simulated microgravity and suggests potential molecular targets for further investigation.

### 2.4. PIEZO1 Upregulates HMGA2 Expression Through Ca^2+^ Influx in Endothelial Cells Under Simulated Microgravity

**Figure 4:**
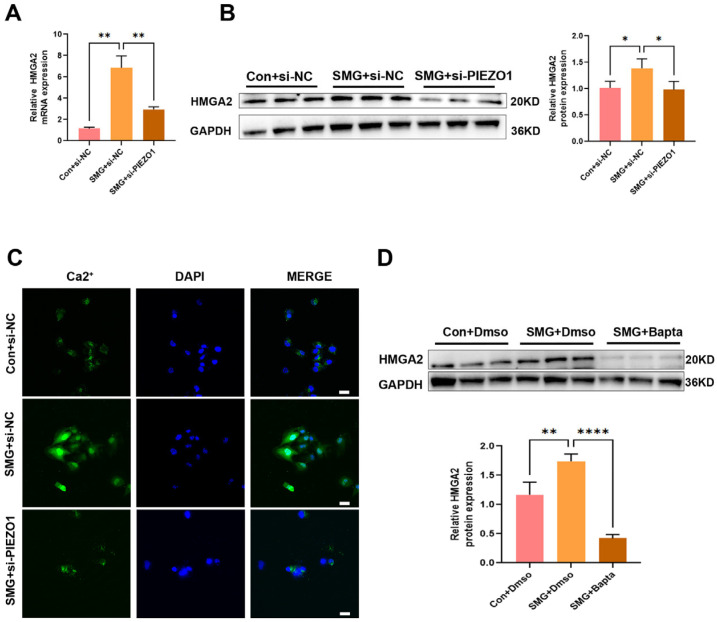
PIEZO1 triggers an increase in HMGA2 expression through the influx of Ca^2+^. (**A**,**B**) Following 48 h of microgravity exposure, the expression of HMGA2 in HUVECs, with or without si-PIEZO1, was analyzed using qRT-PCR and Western blot. (**C**) Confocal microscopy and Immunofluorescence quantification assay were as used to detect Ca^2+^ influx. Ca^2+^ was stained with green immunofluorescence, while DAPI was stained with blue immunofluorescence. (Scale Bar = 100 μm). (**D**) The expression of HMGA2 in HUVECs, with or without BAPTA, was analyzed using Western blot after 48 h of simulated microgravity exposure. (The data represent the mean ± *SEM*, *n* = 3; ns (no significance), *p* > 0.05; * *p* < 0.05, ** *p* < 0.01, **** *p* < 0.0001).

To substantiate the proteomic data indicating that protein PIEZO1 facilitates the upregulation of HMGA2 expression under microgravity conditions and its downregulation following si-PIEZO1 treatment, we performed Western blot analyses. The results confirmed that HMGA2 expression was elevated in response to simulated microgravity and significantly reduced upon siRNA-mediated depletion of PIEZO1 (Figure 4A,B). Ca^2+^ acts as the key mediator for PIEZO1 in the cardiovascular system, whereby its influx enables PIEZO1’s regulatory functions [18]. Given that Ca^2+^ acts as a crucial intracellular second messenger in various cellular processes, we hypothesized that PIEZO1’s role in HMGA2 expression might be linked to its function as an ion channel, facilitating Ca^2+^ influx in response to mechanical stimuli.To evaluate this hypothesis, intracellular Ca^2+^ levels were assessed in HUVECs using Fluo-4 AM fluorescence staining. The results demonstrated a significant elevation in Ca^2+^ concentrations following exposure to simulated microgravity, which was markedly attenuated upon knockdown of PIEZO1 (Figure 4C). To further assess the functional involvement of Ca^2+^ in this regulatory pathway, we employed BAPTA-AM, a cell-permeable calcium chelator, to disrupt Ca^2+^-dependent signaling. Pretreatment with BAPTA-AM substantially suppressed the upregulation of HMGA2 protein expression induced by simulated microgravity (Figure 4D). Collectively, these findings demonstrate that PIEZO1 promotes HMGA2 expression through Ca^2+^ influx in conditions of simulated microgravity.

Our findings elucidate a PIEZO1-to-HMGA2 pathway mediated by Ca^2+^ signaling in HUVECs under simulated microgravity, uncovering a novel mechanosensitive pathway for gene expression regulation in endothelial cells.

### 2.5. HMGA2 Silencing Attenuates Apoptosis in Endothelial Cells Under Simulated Microgravity

**Figure 5:**
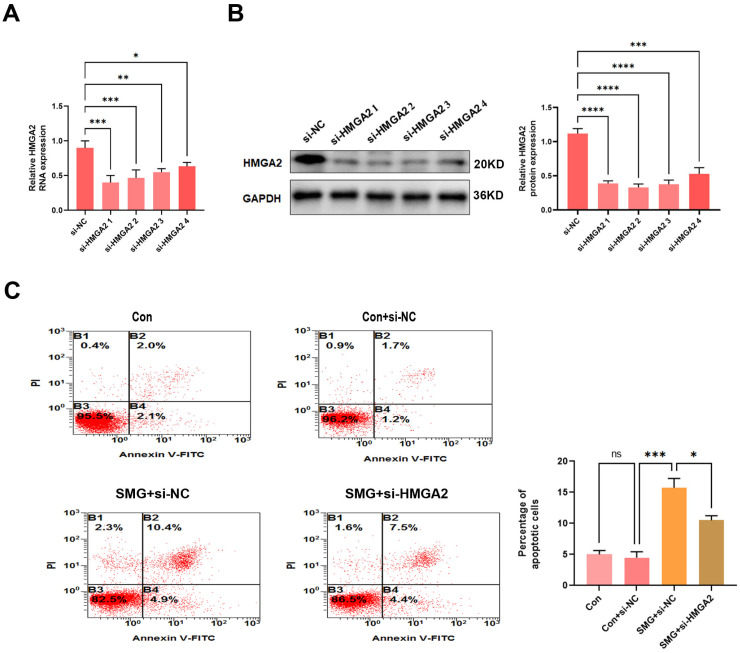
HMGA2 promotes apoptosis in HUVECs under simulated microgravity. (**A**,**B**) Knockdown efficiency of HMGA2 in HUVECs was assessed by qRT-PCR (**A**) and Western blot analysis (**B**) following HMGA2 siRNA transfection. (**C**) Apoptosis of HUVECs was evaluated by flow cytometry using Annexin V-FITC and propidium iodide (PI) staining. (The data represent the mean ± *SEM*, *n* = 3; * *p* < 0.05, ** *p* < 0.01, *** *p* < 0.01, **** *p* < 0.0001).

To explore whether the molecular mechanism underlying HMGA2-mediated apoptosis of HUVECS under simulated microgravity, we conducted the cell apoptosis assay in HUVECs. Initially, we achieved efficient HMGA2 knockdown using an optimized small interfering RNA (siRNA) protocol, with the most effective construct validated through quantitative reverse transcription PCR (qRT-PCR) and Western blot analysis (Figure 5A,B). We observed that exposure to simulated microgravity for 48 h increased apoptosis in HUVECs. HMGA2 silencing led to a partial attenuation of this microgravity-induced pro-apoptotic response compared to the SMG+ si-NC group, indicating that HMGA2 contributes to endothelial apoptosis under simulated microgravity conditions. (Figure 5C). Together, these results indicate that HMGA2 facilitates apoptosis likely through the regulation of apoptosis-related protein expression.

## 3. Discussion

This study elucidates a novel mechanotransduction pathway in which PIEZO1 mediated Ca^2+^ influx regulates ECs apoptosis via HMGA2 signaling under simulated microgravity. We first provide experimental evidence that microgravity exposure upregulates PIEZO1 expression, subsequently enhancing HMGA2 expressions in a Ca^2+^-dependent manner.

Exposure to microgravity eliminates hydrostatic pressure gradients within the vasculature, leading to a cephalad redistribution of body fluids from the lower extremities to the upper body [19]. This fluid redistribution is thought to be the initial trigger for cardiovascular dysfunction, which is highly likely rooted in endothelial dysfunction [20]. Microgravity conditions alter mechanical stress distribution, leading to significant cardiovascular adaptations, manifested as impaired cardiac function and vascular remodeling [21,22]. Vascular endothelial cells, which form the inner lining of blood vessels, experience critical mechanical stimuli including fluid shear stress, circumferential stretch, and transmural pressure. These biomechanical forces are fundamental regulators of endothelial function and vascular remodeling [23]. Piezo1 has been identified as a mechanosensitive ion channel that regulates endothelial cell function. As a Ca^2+^-permeable nonselective cation channel, Piezo1 can be activated by fluid shear stress and has been shown to mediate exercise-induced blood pressure elevation in mice, demonstrating its crucial role in translating mechanical stimuli into endothelial responses [24,25]. Our qRT-PCR and Western blot analysis confirmed the upregulation of PIEZO1 under simulated microgravity and revealed its modulatory action.

Apoptosis is a key mechanism driving vessel regression under conditions of vessel network involution, such as hyaloid vessel regression [26] and retinal vessel obliteration in the oxygen-induced retinopathy model [27]. During mouse retina angiogenesis, the majority of apoptotic endothelial cells ECs are predominantly located in regressing vessels [11]. Notably, vessel non-perfusion precedes ECs apoptosis in hyperoxia-induced vaso-obliteration, with significant apoptosis observed in non-perfused hyaloid vessels. As retinal vasculature matures, ECs apoptosis becomes more widespread [17]. Given that mechanical stimulation can induce endothelial cell apoptosis, it follows that simulated microgravity, as a form of such stimulation, also triggers this effect [28]. Our previous work demonstrated that simulated microgravity induces ECs apoptosis, while miR-27b-5p confers protection by targeting ZHX1 [7]. Intriguingly, recent studies implicate PIEZO1 as a regulator of apoptosis across cell types. For instance, PIEZO1 sensitizes cells to TRAIL-mediated apoptosis by increasing mitochondrial outer membrane permeability [28], and it also promotes chondrocyte apoptosis [29,30]. In this experiment, flow cytometry revealed that PIEZO1 significantly increased the apoptosis rate of HUVECs under simulated microgravity (Figure 2C). In mammalian cells, Bcl-2 exerts its pro-survival function by inhibiting mitochondrial cytochrome c release under various apoptotic stimuli [31]. Bax counteracts the antiapoptotic effect of Bcl-2 by forming Bax/Bax homodimers, thereby promoting cell apoptosis [32]. Hence, we investigated the expression levels of Bax and Bcl-2 in HUVECs under simulated microgravity. Under simulated microgravity, PIEZO1 activation counteracted the upregulation of pro-apoptotic Bax, while its knockdown rescued the downregulation of anti-apoptotic Bcl-2. To our knowledge, this is the first evidence that PIEZO1 modulates microgravity-induced ECs apoptosis via Bax/Bcl-2 signaling.

In recent years, proteomics sequencing has emerged as a powerful tool in cardiovascular research [33]. Advances in mass spectrometry have significantly enhanced our ability to identify and quantify proteins in complex biological samples, thereby providing novel insights and approaches for the investigation of cardiovascular diseases [34]. In the present study, we employed mass spectrometry to elucidate the effects of PIEZO1 on endothelial cell function under microgravity conditions. In the present study, we employed mass spectrometry to elucidate the effects of PIEZO1 on endothelial cell function under microgravity conditions. Our proteomic analysis revealed that PIEZO1 modulates several critical signaling pathways involved in endothelial cell function, including protease binding, cytokine receptor binding and iron ion binding. Notably, we observed that HMGA2, a nonhistone chromatin protein containing three AT-hook DNA-binding motifs, was upregulated under simulated microgravity conditions. HMGA2 binds to the minor groove of AT-rich DNA sequences and regulates transcription by altering chromatin architecture. Conversely, PIEZO1 knockdown resulted in downregulation of HMGA2, suggesting that PIEZO1 may play a role in modulating HMGA2 expression. Our findings highlight the potential involvement of HMGA2 in apoptosis signaling pathways under these conditions.

HMGA2 has been shown to regulate cellular apoptosis, with its effects varying depending on the cell type. For instance, HMGA2 can inhibit apoptosis in cancer cells [35], while it induces apoptosis in primary human fibroblast cells [36]. In this study, we discovered that PIEZO1 regulates HMGA2 expression through Ca^2+^ influx. To elucidate the role of HMGA2 in mediating apoptosis of ECs under simulated microgravity, we performed flow cytometry analysis. Our results demonstrated that simulated microgravity induces apoptosis in ECs, an effect that is significantly alleviated by HMGA2 knockdown. This finding suggests a novel mechanism by which PIEZO1 acts as a mediator for HMGA2-induced apoptosis in ECs under simulated microgravity.

Despite the above findings, several limitations should be considered in the context of recent advances in endothelial mechanobiology. The PIEZO1–Ca^2+^–HMGA2 axis identified here should be interpreted as a contributing rather than exclusive pathway: PIEZO1 upregulation under simulated microgravity may reflect a multifactorial mechanical context, and future studies with appropriate mechanical controls are needed to establish causality; additionally, Ca^2+^ elevation in microgravity could involve other mechanosensitive channels and/or altered intracellular Ca^2+^ handling. Although HMGA2 knockdown attenuated apoptosis, it remains to be determined whether HMGA2 acts as a direct pro-apoptotic transcriptional regulator or functions more broadly as a chromatin remodeling factor that sensitizes ECs to microgravity-associated stress, which warrants dedicated mechanistic studies.

## 4. Materials and Methods

### 4.1. Cell Culture and Drug Treatment

Human umbilical vein endothelial cells (HUVECs) were obtained from the American Type Culture Collection (ATCC, USA, Cat. CRL1730) and maintained in high-glucose DMEM (Hyclone, Logan, UT, USA) containing 10% fetal bovine serum (FBS, Hyclone). Cells were grown under standard culture conditions (37 °C, 5% CO_2_, humidified atmosphere), with media refreshed at 48 h intervals to maintain viability. Subculturing was performed using 0.25% trypsin-EDTA upon reaching 80% confluency to prepare cells for downstream experiments. For calcium quantification, intracellular Ca^2+^ levels were assessed using Fluo4-AM (5 µM final concentration; Beyotime, Shanghai, China), a membrane-permeable fluorescent probe. To control for baseline calcium interference, selected cell groups were pre-treated with the calcium chelator BAPTA-AM (10 µM; MedChemExpress, Monmouth Junction, NJ, USA), dissolved in dimethyl sulfoxide (DMSO), for 30 min prior to experimentation.

### 4.2. Clinorotation to Simulate Microgravity

The two-dimensional clinorotation system (China Astronaut Research and Training Center, Beijing, China), an established protocol for ground-based microgravity simulation [1,2], was employed in this study. HUVECs were plated at 1 × 10^5^ cells/cm^2^ on sterile glass coverslips (2.55 × 2.15 cm) within six-well plates and allowed to adhere under standard culture conditions (37 °C, 5% CO_2_). Following adhesion stabilization, coverslips were aseptically transferred to custom-designed clinorotation chambers filled with complete growth medium. The microgravity group (MG) underwent continuous axial rotation at 30 rpm in the clinostat, generating sustained gravitational forces below 10^−3^ g—a validated model for mimicking spaceflight conditions [1]. Parallel control cultures (Con) were maintained under static conditions within identical chambers. All experimental groups were processed simultaneously under temperature-controlled (37 °C) protocols to ensure comparability.

### 4.3. Flow Cytometry

After treatment, cell samples were harvested by centrifugation at 1000 rpm for 5 min. Subsequently, the cell pellets were resuspended and stained with 5 μL Annexin V-FITC and 5 μL propidium iodide (PI) at ambient temperature (20–25 °C). Following the manufacturer’s protocol, the samples were incubated in light-protected conditions for 15 min. Cellular apoptosis was quantitatively assessed using a FACS Calibur flow cytometer (Becton Dickinson, NJ, USA), with all analyses completed within 60 min of staining completion.

### 4.4. qRT-PCR Analysis

To analyze the expression levels of target genes in human umbilical vein endothelial cells (HUVECs), total RNA was first extracted using TRIzol Reagent (Invitrogen, Carlsbad, CA, USA) following the manufacturer’s instructions. A quantity of 0.5 µg of the isolated RNA was then reverse-transcribed into cDNA using the PrimeScript RT reagent Kit (Takara, Tokyo, Japan). Real-time PCR was subsequently performed with SYBR^®^ Premix Ex Taq™ II (YISHEN, Shanghai, China) on a Roche LightCycler 480 system (Roche, Manheim, Germany). The PCR cycling conditions included an initial denaturation step at 95 °C for 30 s, followed by 40 cycles consisting of denaturation at 95 °C for 5 s and annealing/extension at 60 °C for 40 s. The endogenous reference gene GAPDH was employed for data normalization. The relative mRNA expression levels were determined using the 2−∆∆Ct method and are presented as fold changes relative to GAPDH. The primer sequences used were as follows: for human PIEZO1, forward primer 5′-CCTGGAGAAGACTGACGGCTAC-3′ and reverse primer 5′-ATGCTCCTTGGATGGTGAGTCC-3′; for human GAPDH, forward primer 5′-GTCTCCTCTGACTTCAACAGCG-3′ and reverse primer 5′-ACCACCCTGTTGCTGTAGCCAA-3′; for human HMGA2, forward primer 5′-AccCAggggAAgACcCAAA-3′ and reverse primer 5′-cCTCTTggcCgTTTTTCTCCA-3′.

### 4.5. Western Blot Analysis

To isolate total protein from cells, we utilized RIPA buffer (NCM Biotech, Suzhou, China) containing 1 mM phosphatase inhibitor cocktail, conducting the extraction on ice. The protein concentration was subsequently determined using the BCA assay kit (Beyotime, Shanghai, China). The protein samples were then denatured by boiling in loading buffer (Bio-Rad, Hercules, CA, USA) for 10 min. After denaturation, the samples underwent sodium dodecyl sulfate-polyacrylamide gel electrophoresis (SDS-PAGE) and were transferred onto a 0.45 µm polyvinylidene fluoride (PVDF) membrane. PIEZO1 was separated by electrophoresis on a 6% polyacrylamide gel, while the other proteins were analyzed on 4–20% SurePAGE precast gradient gels (M00657, GeneScript, Nanjing, China). The PVDF membranes were blocked with 5% non-fat milk in Tris-buffered saline-Tween 20 (TBST) at room temperature for 2 h. Following blocking, the membranes were incubated overnight at 4 °C with primary antibodies: anti-PIEZO1 (1:1000, Abcam, Cambridge, UK, ab259949), anti-HMGA2 (1:2000, Proteinteach, Wuhan, China, 20795-1-AP),anti-BcL-2(1:1000, Selleck, Houston, TX, USA, F0125), anti-BAX (1:5000, Proteinteach, Wuhan, China, 60267-1-Ig) and anti-GAPDH (1:2000, Zhuangzhi Biology, Xi’an, China, NC021). Subsequently, the membranes were treated with horseradish peroxidase (HRP)-conjugated secondary antibody for 1 h at room temperature. The protein bands were detected using an enhanced chemiluminescence (ECL) system (Millipore, Danvers, MA, USA). The intensity of the bands was assessed with ImageJ software (ImageJ Fiji 1.54f.) and normalized to GAPDH, which acted as a loading control.

### 4.6. Immunofluorescence Staining

Cells were first treated with a 4% paraformaldehyde solution for 10 min and then permeabilized with 0.5% Triton X-100 in PBS at room temperature for 30 min. To reduce non-specific binding, the cells were blocked with 10% normal goat serum. Subsequently, the cells were incubated overnight at 4 °C with a PIEZO1 antibody (1:200; Proteintech, Wuhan, China). On the following day, the cells were treated with an Alexa Fluor 488-conjugated secondary antibody (1:1000; Beyotime, Shanghai, China) for 1 h at room temperature. Nuclei were stained with DAPI in the dark. The fluorescence was visualized using CaseViewer 2.4 software (3DHISTECH, Budapest, Hungary).

### 4.7. Transfection of Small Interfering RNA

For RNA interference experiments, cells were transfected with PIEZO1-specific siRNA (si-PIEZO1), HMGA2-specific siRNA(si-HMGA2) and negative control siRNA (si-NC) at 70% confluence using Lipofectamine 2000 (Invitrogen, USA) following the manufacturer’s instructions. The sequences of the siRNA probes were as follows: si-NC, 5′-UUCUCCGAACGUGUCACGUTT-3′; si-PIEZO1, 5′-CACCGGCATCTACGTCAAATA-3′; si-HMGA2, 5′-GAGACAUCCUCACAAGAGUTT-3′. Transfection efficiency was assessed by qPCR and Western blot analysis 48 h post-transfection.

### 4.8. Cytosolic Ca^2+^ Measurements

For Ca^2+^ imaging experiments, we used Fluo4-acetoxymethyl ester (Fluo4-AM, Beyotime, Shanghai, China). Cells were exposed to 5 µM Fluo4-AM in a Hanks’ balanced salt solution (HBSS; 0.137 M NaCl, 5.4 mM KCl, 0.25 mM Na_2_HPO_4_, 0.44 mMKH_2_PO_4_, 4.2 mM NaHCO_3_, 5.56 mM glucose, and 10 mM HEPES, pH 7.4) lacking Ca^2+^ and Mg^2+^ for 30 min at room temperature. After incubation, the cells were washed twice with HBSS to remove excess dye. Fluorescence measurements were then taken using an LSM 800 confocal fluorescence microscope (Zeiss, Oberkochen, Germany). At 488 nm, Fluo-4 was excited, and the fluorescence emitted was captured using a barrier filter between 505 and 550 nm. The images were then processed and analyzed with ImageJ software (https://imagej.net/ij/index.html, accessed on 25 June 2024). The data were normalized to baseline fluorescence for comparative analysis.

### 4.9. Proteomic Analysis

#### 4.9.1. Sample Preparation

Protein lysates were generated from human umbilical vein endothelial cells (HUVECs) across three experimental groups—Con+si-NC, SMG+si-NC, and SMG+si-PIEZO1—with three biological replicates per group. Cells were lysed in a buffer composed of 8 M urea, 1% protease inhibitor, and 1% phosphatase inhibitor. Lysates were sonicated for 3 min on ice to ensure complete disruption, followed by centrifugation at 12,000× *g* for 10 min at 4 °C to remove cellular debris. The supernatant was transferred to a fresh tube, and protein concentration was determined using a BCA assay kit. For subsequent enzymatic digestion, equal amounts of protein from each sample were adjusted to uniform volume with lysis buffer. Proteins were precipitated by adding trichloroacetic acid (TCA) to a final concentration of 20%, vortexing gently, and incubating at 4 °C for 2 h. The samples were centrifuged at 4500× *g* for 5 min, and the supernatant was discarded. Protein pellets were washed 2–3 times with pre-chilled acetone to remove impurities and then air-dried for 1 min before being resuspended in 200 mM triethylammonium bicarbonate (TEAB). Digestion was performed overnight with trypsin added at a 1:50 (enzyme: protein, *w*/*w*) ratio. Dithiothreitol (DTT) was used to reduce disulfide bonds by incubating the samples at 56 °C for 30 min at a final concentration of 5 mM. Iodoacetamide (IAA) was subsequently added to a final concentration of 11 mM, and the samples were incubated in the dark at room temperature for 15 min to alkylate cysteine residues. Finally, peptides were purified using a Strata X solid-phase extraction column to remove salts and other contaminants.

#### 4.9.2. Liquid Chromatography and Tandem Mass Spectrometry (LC–MS/MS)

Peptides were dissolved in solvent A and directly injected onto a custom-made reversed-phase analytical column (25 cm in length, 100 µm inner diameter). The mobile phase consisted of solvent A (0.1% formic acid and 2% acetonitrile in water) and solvent B (0.1% formic acid and 90% acetonitrile in water). Peptides were separated using a gradient elution: 6–22% solvent B over 0–22.5 min, 22–34% solvent B over 22.5–26.5 min, 34–80% solvent B over 26.5–28.5 min, and 80% solvent B from 28.5 to 30 min. This separation was performed at a constant flow rate of 700 nL/min using an EASY-nLC 1200 UPLC system (ThermoFisher Scientific, Waltham, MA, USA). The separated peptides were analyzed by nano-electrospray ionization on an Orbitrap Exploris 480 mass spectrometer. The FAIMS compensation voltage (CV) was set to 45 V, and the electrospray voltage was adjusted to 2300 V. The Orbitrap detector was employed for both precursor and fragment analysis. The full MS scan was conducted over a mass range of 350–1400 *m*/*z* with a resolution of 60,000. For MS/MS scans, the first mass was fixed at 120.0 *m*/*z* with a resolution of 15,000. The normalized collision energy (NCE) for HCD fragmentation was set to 27%. The automatic gain control (AGC) target was set to 10^6^, with an injection time of 22 ms.

#### 4.9.3. Data Processing

To construct the Spectral Library, data from Data Dependent Acquisition (DDA) were processed using Spectronaut (v.17.0) software in conjunction with the Pulsar search engine. The tandem mass spectra were matched against the Homo_sapiens_9606_SP_20230103 fasta database (20,389 entries), which was concatenated with reverse decoy sequences. The maximum number of missed cleavages was set to 2. The false discovery rates (FDR) for peptide-spectrum matches (PSM), peptides, and proteins were all controlled at 1%. In Spectronaut (v.17.0) software, the corresponding spectral library was imported, and retention times were predicted using nonlinear correction. These predicted retention times were then compared with Data Independent Acquisition (DIA) data. Specifically, the spectral library was used to search against DIA data within Spectronaut (v.17.0) to facilitate accurate peptide identification and quantification.

#### 4.9.4. Bioinformatics Analysis

For bioinformatics analysis, clustering was performed using the “Mfuzz” R package (version 3.2.3). Gene ontology (GO) and pathway analysis were conducted using the eggnog-mapper software-1.0.3 (https://anaconda.org/bioconda/eggnog-mapper, accessed on 19 June 2025) to retrieve GO IDs from the identified proteins in the EggNOG database. The functional enrichment significance of differentially expressed proteins (DEPs) was evaluated using Fisher’s exact test. Enrichment values with a fold change greater than 1.5 and a *p*-value less than 0.05 were considered statistically significant.

### 4.10. Statistical Analysis

Statistical analyses were performed using GraphPad Prism software (version 8.3.0). Data are presented as the mean ± SEM from three independent experiments. For multiple comparisons involving more than two normally distributed groups, one-way ANOVA followed by Dunnett’s post-test was applied. *p* < 0.05 was considered to indicate statistical significance.

## 5. Conclusions

In summary, this study identifies a mechanotransduction pathway in which simulated microgravity activates PIEZO1, leading to increased Ca^2+^ influx and subsequent upregulation of HMGA2, thereby contributing to endothelial cell apoptosis. These findings provide new mechanistic insight into how altered mechanical environments may promote endothelial dysfunction under simulated microgravity conditions. However, the PIEZO1–Ca^2+^–HMGA2 axis described here should be interpreted as a contributing rather than exclusive mechanism, as endothelial Ca^2+^ signaling and apoptotic regulation under microgravity are likely influenced by multiple mechanosensitive pathways. Future studies employing refined mechanical controls and complementary experimental models will be essential to fully define the causal relationships and physiological relevance of this pathway.

## Data Availability

The data that support the findings of this study are available from the corresponding authors upon reasonable request.

## Supplementary Materials

The following supporting information can be downloaded at: https://www.mdpi.com/article/10.3390/ijms27031425/s1.
